# Biomarkers of aging: functional aspects still trump molecular parameters

**DOI:** 10.1038/s41514-025-00207-2

**Published:** 2025-03-03

**Authors:** Regula Furrer, Christoph Handschin

**Affiliations:** Biozentrum, University of Basel, Basel, Switzerland

**Keywords:** Biomarkers, Ageing

## Abstract

Biomarkers of aging are indispensable for testing interventions. While promising, the recent focus on molecular aspects should not detract from the functional parameters for which excellent correlation with mortality, and ample clinical human data exist.

The almost global shift towards aging population demographics has triggered an enormous push to better understand and potentially counteract not only aging-associated diseases, but also aging as a process. Even though the underlying mechanisms are still mysterious, several interventions and pharmacological treatments have already been proposed based on mostly pre-clinical data in rodents and other model organisms. Translation to humans however is hampered by the long duration of clinical trials with the endpoint mortality due to the high longevity of our species, as well as the uncertainty whether aging can be considered as a bona fide disease or rather represents a normal biological program. While the latter question remains to be resolved, clinical trials could be shortened to reasonable time frames if biomarkers that accurately reflect the consequences of aging (and the effects of treatments) were available. Different types of molecular events have been put forward to serve as such biomarkers. However, consensus on the validity and use of molecular aging biomarkers has not emerged yet, as most of these marks are associative, and very few, if any, have been conclusively tested in humans. It thus is somewhat surprising that other biomarkers of functional capacity, morbidity, and mortality, centered on (multi-)organ function and anthropometry, for which excellent clinical data exist^1^, are somewhat underappreciated and hardly discussed in contemporary literature on the topic of biomarkers of aging. In this commentary, we briefly describe the state-of-the-art of some of the molecular and physiological biomarkers, the limitations and advantages, and how we could move forward by reconciling the use of all of these. Moreover, we explain how the physiological biomarkers are intrinsically linked to the current interventions for which solid human data in improving morbidity and lifespan has been provided. Due to space constraints, we however cannot provide a comprehensive and systematic review. For this, readers are referred to review articles, e.g., ref. ^2^.

## Molecular biomarkers of aging

The first “aging clocks” were proposed based on the observation of changing epigenetic marks during development (Waddington’s model of epigenetic determination), a concept which was subsequently extrapolated to aging^3^. Accordingly, epigenetic marks, e.g., in DNA methylation, could serve as an indicator of “biological age” that can coincide or diverge in either direction from “chronological age”. Whether the corresponding epigenetic events are deterministic or stochastic is unclear for most of the proposed epigenetic “clocks”, as are the potential functional consequences. Other markers include telomere length, transcriptomic fingerprints, metabolome- and protein-/glycan-based “age scores” or human plasma proteome profiles. With few exceptions, these “clocks” have primarily been tested in rodent models or small human groups, and require technically demanding procedures. In recent years, human translatability has been tested in larger cohorts, most notably for plasma proteome profiles (e.g., refs. ^4,5^). Of note, when tested concomitantly, the “clocks” show little correlation and overlap, and can vary between organs, cell types, and even the same cell type depending on spatial location within an organ, and might oscillate in a circadian manner^3,6^. To improve this situation and accelerate the future development of robust biomarkers of aging, guidelines for the identification, evaluation, and validation have been proposed^7–9^. For reasons unknown, these guidelines however primarily refer to molecular biomarkers of aging, while largely neglecting other types of biomarkers that are much more advanced in clinical application.

## Physiological biomarkers of aging, morbidity, and mortality

**Fig. 1:**
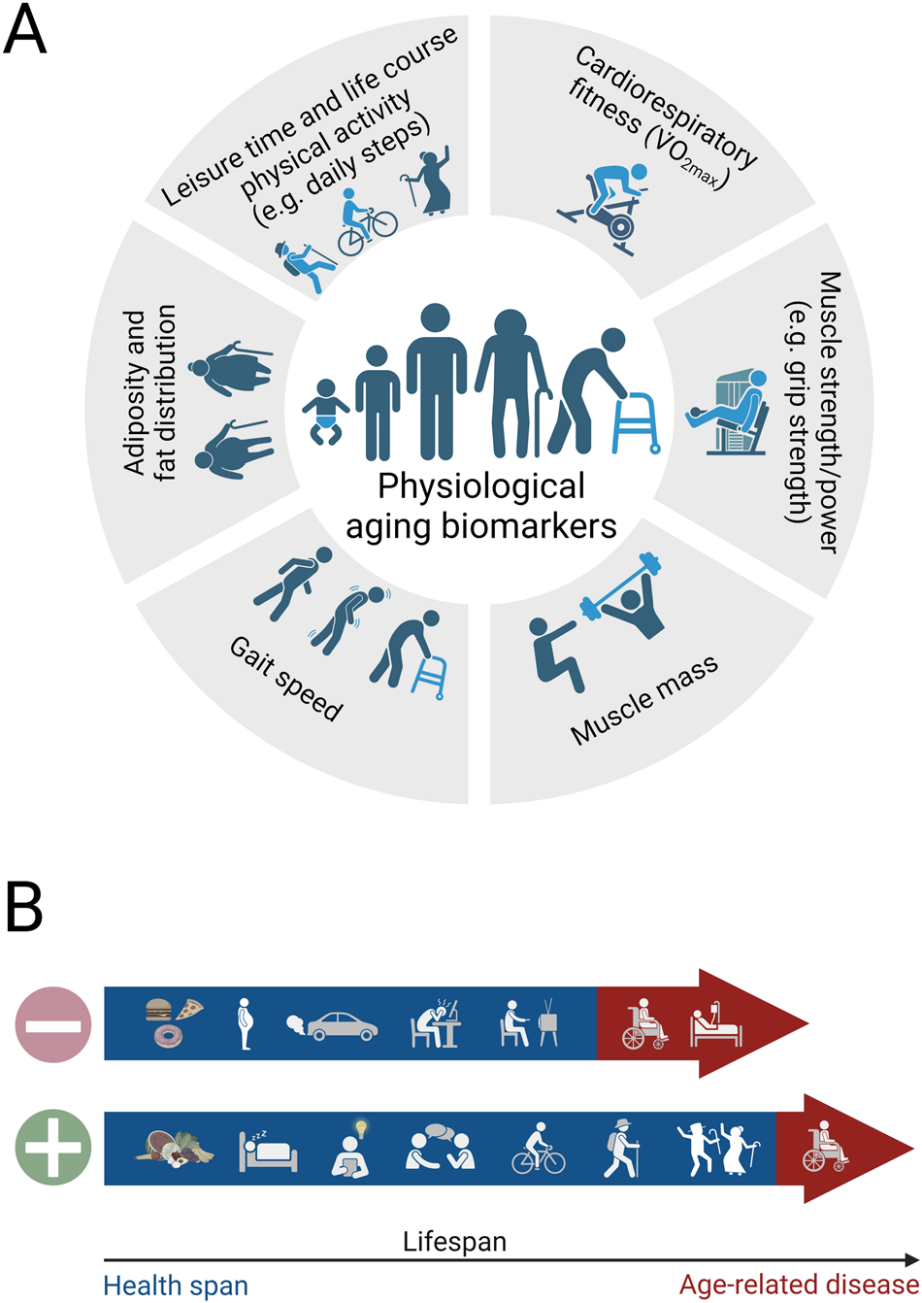
Physiological biomarker of aging, morbidity, and mortality. **A** Examples of physiological biomarkers with established correlation with human longevity, morbidity, and mortality. Most of these physiological markers are centered on cardiorespiratory fitness, skeletal muscle strength, neuromuscular function, and muscle, as well as fat mass and distribution. **B** Examples of interventions and lifestyle choices with a proven effect on human health span, longevity, morbidity, and mortality. The beneficial behavioral choices include a balanced and calorically controlled diet, adequate sleep, intellectual and cognitive challenges, social interactions, physical activity, and exercise. Figure created with Biorender.com.

Inverse to the bottom-up strategy of identifying molecular biomarkers based on correlative molecular events from model organisms to humans, physiological biomarkers of aging, morbidity, and mortality have emerged from clinical tests in vast human cohorts (e.g., refs. ^10–12^) (Fig. 1A). Moreover, in contrast to the unknown causality of the events underlying molecular biomarkers to aging progression and pathology, physiological biomarkers provide a direct readout on (multi-)organ health and morphology. For example, one of the best-studied parameters, cardiorespiratory fitness (CRF) provides insights into the functional capacity of the respiratory, cardiac, vascular, and muscular systems, with excellent predictive power on morbidity and mortality in different age groups, both sexes, diverse ethnic backgrounds, healthy and diseased individuals, and other demographic cohorts^13^. CRF is assessed by measuring maximal oxygen uptake (V̇O_2max_), and can be expanded by other tests such as concomitant application of blood pressure measurements or echocardiography, with additional insights into health-relevant aspects. Anthropometric determination of (segmented) lean, or better skeletal muscle mass in relation to fat content and distribution likewise predicts morbidity and mortality to a high degree, much better than the simplified use of the body mass index (BMI)^14^. In recent years, the superior importance of skeletal muscle function over muscle mass has been appreciated, in particular in the etiology and treatment of sarcopenia (loss of muscle mass during the aging process). Dynapenia or Powerpenia, the loss of muscle strength or power, respectively, might thus be better descriptors of age-related muscle decline. Accordingly, muscle strength, representing maximal force in a single contraction for example measured in the hand grip, and power, representing force production over time, predict mortality, with a higher relevance for the estimation of residual functional capacity compared to muscle mass^15^. All of these parameters, CRF, muscle mass, and function are directly and inversely affected by exercise training and a sedentary lifestyle. Hence, the amount of leisure time and life-course physical activity are highly correlated with morbidity and mortality^16^. Increasingly sophisticated wearables help to overcome the limitations of self-reporting in assessing physical activity. Nevertheless, even a simple parameter such as daily step count is predictive of mortality^17^. Inadequate engagement in physical activity has consequences beyond reduced muscle function, greatly exacerbating decline in multi-organ function and as a consequence, resulting in a frail phenotype. It thus is of little surprise that neuromuscular function, e.g., expressed in gait speed, and other frailty-related parameters, often used as compound scores indicative of a general functional decline, also strongly correlate with mortality^18^.

## Current advantages of physiological biomarkers of aging

These and other physiological biomarkers currently have clear advantages over molecular biomarkers of aging. First, human relevance has been established in very large cohorts of different demographics, with excellent predictive power for morbidity and mortality. Second, the corresponding tests are standardized, can be adapted to accommodate clinical populations, do not necessitate highly specialized knowledge for execution and interpretation, and are minimally invasive and inexpensive. Third, inherent causality of the outcome of the physiological biomarker measurements in regard to functional capacity in aging and age-related pathologies can be established. Fourth, the physiological biomarkers are pliable by interventions for which proof of beneficial effects on the aging process has been established in humans. In contrast, these points are mostly lacking for molecular biomarkers of aging, some of which might measure different types of transient stress instead of or in addition to bona fide aging^19^. Very few limitations for the acquisition and interpretation of physiological biomarkers exist: even for the more physically demanding measurement of V̇O_2max_, guidelines for tests in vulnerable individuals, e.g., elderly people or cardiac patients, have been successfully tested and applied (e.g., ref. ^20^).

## “Anti-aging” interventions and biomarkers of aging

In recent years, various interventions, e.g., caloric restriction, and pharmacological agents, e.g., resveratrol, metformin, NAD+ precursors, or rapamycin, have been proposed to exert “anti-aging” effects. At the moment, only preclinical data exist, and, with the exception of rapamycin, in most cases, the effects are not reproducible on a broad scale in model organisms, even for the “gold standard” caloric restriction^21^. In contrast, a number of modifiable lifestyle-based interventions exhibit a strong correlation with longevity and health in humans (Fig. 1B), including social interactions, intellectual and cognitive challenges, a balanced and calorically-controlled diet, or adequate sleep^6,22^. Other factors with similar predictability might be more difficult to change, such as socioeconomic status or air pollution. The best evidence for pro-life- and -health span effects currently exists for physical activity^6,22^. Exercise training elicits pleiotropic adaptations in almost every organ of the human body^23^, thus on a similar scale compared to the changes observed in the physiological aging process. It is difficult to conceive how pharmacological modulation of single targets or pathways could result in comparable outcomes^24^. Of note, the physiological biomarkers are causally and intrinsically linked to the amount, type, and consequences of exercise. Moreover, a direct read-out of the functional capacity in aging emerges from the physiological biomarkers. Largely, these parameters determine quality of life, degree of independence, or admission to nursing homes, thus tangible and relevant outcomes for the elderly. In stark contrast, the events that are measured in the molecular biomarkers and “aging clocks” are largely correlative, with little information about functional consequences. In fact, physical activity, diet, and sleep are the main recommendations provided by commercial suppliers that offer the determination of an individual's “aging clock” based on molecular biomarkers due to the lack of alternative actionable interventions emerging from the molecular data.

## Summary, conclusion, and perspectives

The current research on molecular mechanisms and biomarkers of aging is important and exciting, and will help to better understand this process in the future once the step from correlation to causality and functional consequence is made. Moreover, if occurring earlier in the aging process (or providing higher sensitivity in acquisition), molecular biomarkers might help to highlight unfavorable health trajectories before the onset of physiological changes, even though many of the latter already provide prognostic insights. These new insights however should not detract from the existing data and methods, which, as outlined above, already have overcome many of the hurdles that molecular biomarkers still will have to tackle. First, the physiological biomarkers should be used to benchmark newly proposed parameters. Second, even more importantly, a greater appreciation and broad application of the established physiological biomarkers of aging, functional capacity, morbidity, and mortality, with proven reliability, safety, and human relevance, could help to accelerate the clinical translation of new findings in the aging field. In fact, the suitability and validity are underlined by the acceptance of at least some of these parameters by regulatory agencies for clinical trials, e.g., those aimed at sarcopenia, or in which frailty is an issue^25^.
